# Supplementary material for: Characterization of new RNA polymerase III and RNA polymerase II transcriptional promoters in the Bovine Leukemia Virus genome
Source: Sci Rep. 2016 Aug 22;6:31125. doi: 10.1038/srep31125 (PMC4992882; doi:10.1038/srep31125)
Supplement: Supplementary Information [file srep31125-s1.pdf]

Characterization of new RNA polymerase III and RNA polymerase II transcriptional promoters in the Bovine Leukemia Virus genome

Short title: New promoters in the BLV genome

Benoit Van Driessche[1,#], Anthony Rodari[1,#], Nadège Delacourt[1], Sylvain Fauquenoy[1], Caroline Vanhulle[1], Arsène Burny[1], Olivier Rohr[2,3,$], Carine Van Lint[1,$,*].

[1] Service of Molecular Virology, Department of Molecular Biology (DBM), Université Libre de Bruxelles (ULB), Gosselies, B-6041, Belgium.

[2] Institut Universitaire de Technologie Louis Pasteur de Schiltigheim, University of Strasbourg, Schiltigheim, F-67300, France.

[3] Institut de Parasitologie et de Pathologie Tropicale, Laboratory of Dynamic of Host-Pathogen Interactions (DHPI), EA7292, University of Strasbourg, Strasbourg, F-67000, France.

* To whom correspondence should be addressed. Tel: +32 2 650 98 07
Fax: +32 2 650 98 00
Email: cvlint@ulb.ac.be

[#,$] These authors contributed equally to this work

a

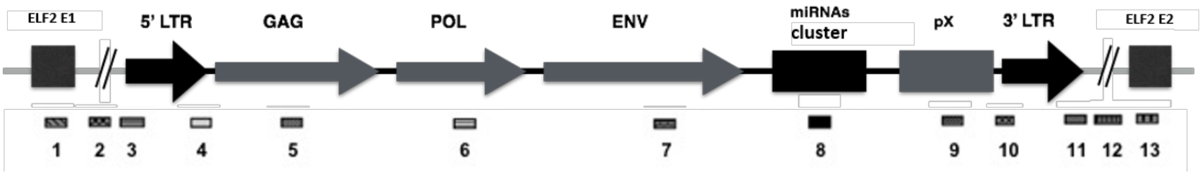

b

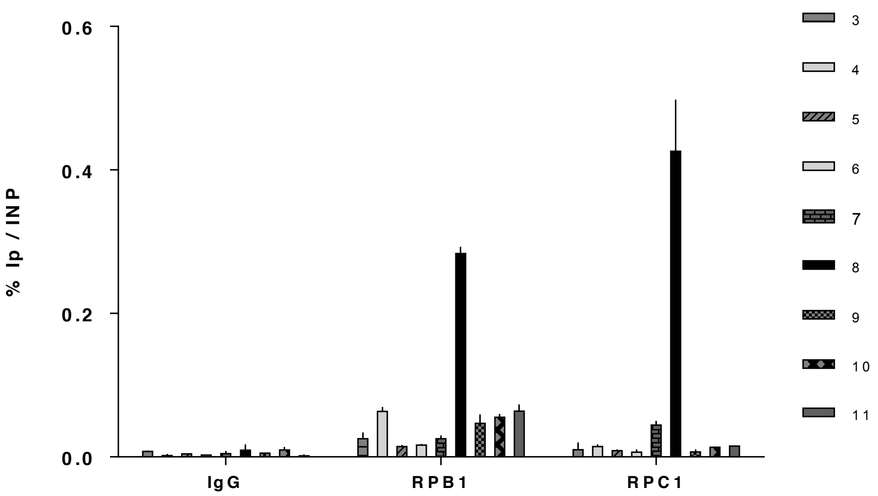

Figure S1. In vivo binding of RNAPIII to the BLV miRNA cluster in latently-infected YR2 ovine cells.
a. Schematic representation of the BLV provirus in latently-infected YR2 ovine cells. The localization of the different amplified regions is presented below the provirus. **b**. Chromatin prepared from YR2 cells was immunoprecipitated with specific antibodies directed against the largest subunit of the RNAPII or RNAPIII (RPB1 or RPC1, respectively) or with a purified IgG to measure aspecific immunoprecipitation background. Results are presented as histograms indicating percentages of immunoprecipitated DNA compared to the input DNA (% IP/INP). Data are the means ± SEM from one representative of at least three independent experiments.

a

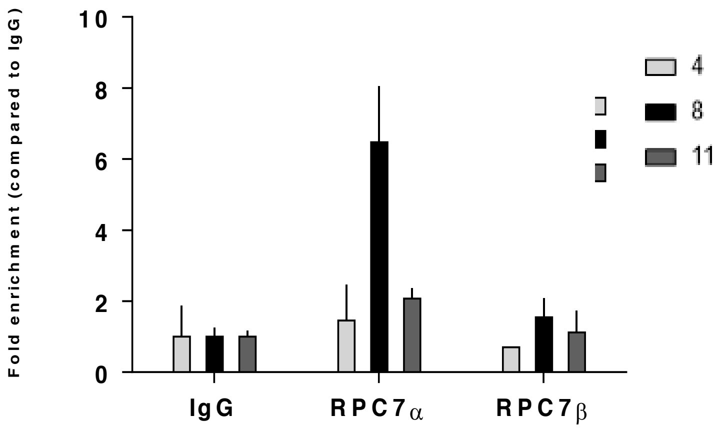

b

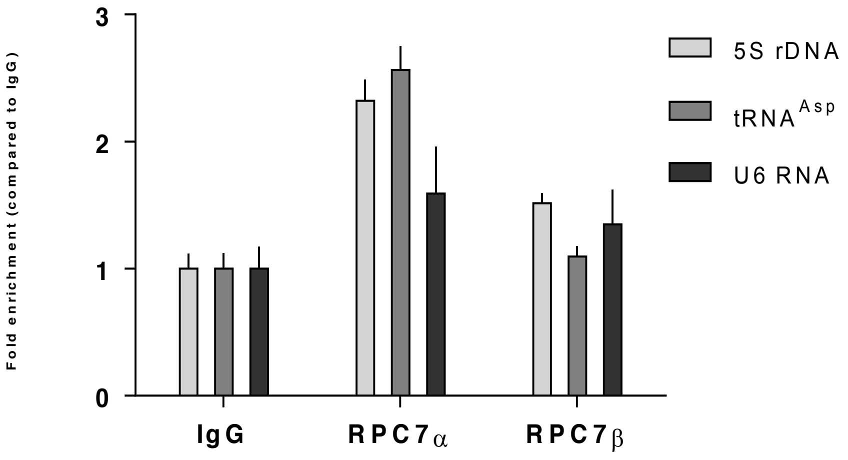

Figure S2. Recruitment of the RPC7α and RPC7b subunits to the BLV miRNA cluster in latently-infected YR2 ovine cells.

Chromatin prepared from YR2 cells was immunoprecipitated with specific antibodies directed against the two isoforms of the RNAPIII RPC7 subunit or with a purified IgG as background measurement. Purified DNA was then amplified with oligonucleotide primers hybridizing to three BLV regions: the 5'-LTR, the miRNA cluster or the 3'LTR/host junction region (**a**) or to the class III control cellular genes (**b**). Results are presented as histograms indicating fold enrichment compared to IgG for which a value of 1 was assigned. Data are the means ± SEM of duplicates samples from one representative of at least three independent experiments.

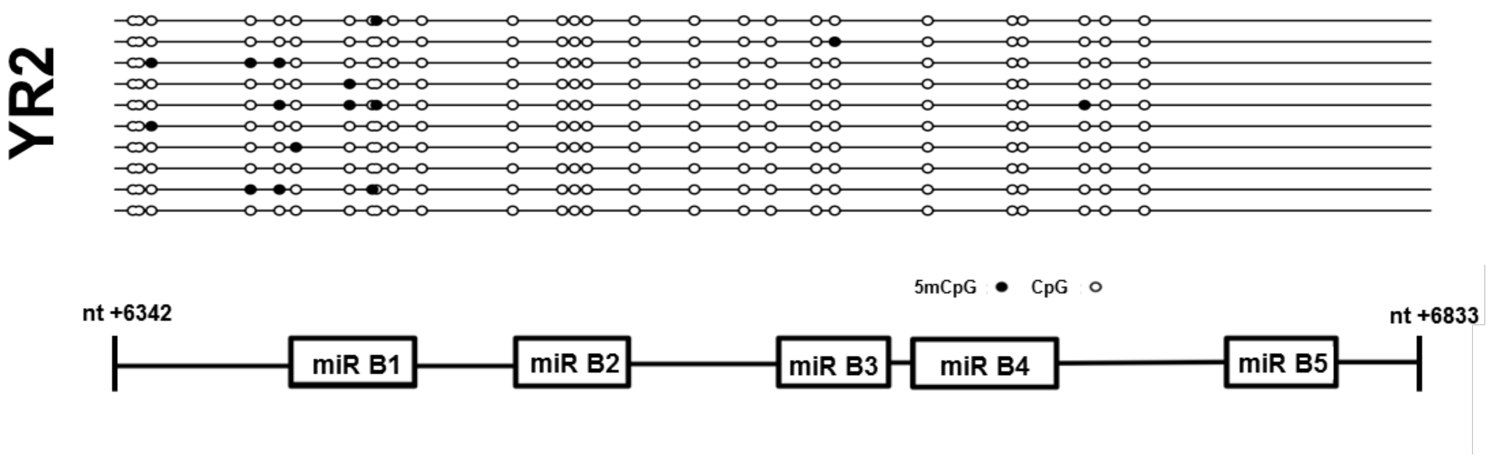

Figure S3. The BLV miRNA cluster DNA is not methylated in latently-infected YR2 ovine cells.
Genomic DNA from YR2 cells was extracted and treated by sodium bisulfite and the miRNA cluster was amplified by nested PCR. The amplified products were cloned in a TA cloning vector and 10 independent clones were sequenced. Open and filled circles represent non-methylated and methylated CpG dinucleotides, respectively. The position of the 5 BLV-pre-miRs within the miRNA cluster is presented in the lower part of the figure.

Name of primer		Sequences
**CV3311**	**Stemloop BLV-miR-B4-3p**	5'…gtcgtatccagtgcagggtccgaggtattcgcactggatacgacaaaggc…3'
**CV3331**	**Stemloop miR-191**	5'…gtcgtatccagtgcagggtccgaggtattcgcactggatacgaccagctg…3'
**CV3333**	**Stemloop BLV-miR-B2-5p**	5'…gtcgtatccagtgcagggtccgaggtattcgcactggatacgactctgcg…3'
**CV3321**	**miRNAs bs Fwd**	5'…tgattatcaggccttgctaccatc…3'
**CV3322**	**miRNAs bs Rev**	5'…gaagggactttatgggcactgttagaa…3'
**CV3323**	**miRNAs bs nested Fwd**	5'…ccagagatctactctcacctctcccc…3'
**CV3324**	**miRNAs bs nested Rev**	5'…gcccaaatgctgctggatgtggctgg…3'
**CV3380**	**miRNAs cluster Fwd**	5'…gtcagcacccccatgtttcacgcacc…3'
**CV3381**	**miRNAs cluster Rev**	5'…gctagcagatctgaccttttggaagagatattg…3'
**CV3451**	**MTE/DPE Fwd**	5'…acaagcgcgggtgcaagccatgattccttggagcgcggggtcagg…3'
**CV3452**	**MTE/DPE Rev**	5'…cctgaccccgcgctccaaggaatcatggtctgcacccgcgcttgt…3'
**CV3453**	**BRE Fwd**	5'…ttgccggtgtctcctggccgctagataacgccgaaggagagagcg…3'
**CV3454**	**BRE Rev**	5'…cgctctctccttcggcgttatctagcggccaggagagaccggcaa…3'

Table S1 : List of PCR primers used in this study

Antibody	Reference	Origin
IgG	I-1000	Vector Laboratories
RPB1	SC-899	Santa Cruz
TBP	Mab-TBPCSH-100	Diagenode
Bdp1	2663	Dr B. White
Brf1	128	Dr B. White
Brf2	940.005	Dr N. Hernandez
TFIIIC	4286	Dr B. White
TFIIIA	ab76894	Abcam
RPC1	CS377	Dr N. Hernandez
RPC1'	SC-1900	Santa Cruz
RPC4	CS681	Dr N. Hernandez
RPC6	ab151495	Abcam
RPC7α	SC3070	Dr N. Hernandez
RPC7β	SC3072	Dr N. Hernandez
H3K27me3	07-449	Millipore
H3K4me2	07-030	Millipore
H3K4me3	04-745	Millipore
H3K36me3	ab-9050	Abcam
H3K9me3	07-442	Millipore
H3ac	06-599	Millipore
H3K9ac	pAb-ACHAHS-044	Diagenode
H2AZ	17-10048	Millipore
α-tubulin	T5168	Sigma

Table S2 : List of antibodies used in this study

Name of primer	Genomic localization	Viral localization (nt +1 = first nucleotide of the 5'LTR)	Sequences
CV3168	*Rasa3 E1*		5'…gacccagcatggcggtggag…3'
CV3169	*Rasa3 E1*		5'…ccgcctgctcctgcgaactc…3'
CV3170	*Rasa3 I1*		5'…catacctccctggccccgct…3'
CV3171	*Rasa3 I1*		5'…cagaagcacccgcgctccaa…3'
CV3174	Jonction 5'		5'…ccggcagcttctgaccgcag…3'
CV3175	Jonction 5'	nt 9 – nt 28 and nt 8198 –nt 8217	5'…cgcctaggccggcatgatct…3'
CV3123	LTR 5'	nt 423 – nt 442 and nt 8612 – nt 8631	5'…aagggcgtctggcttgcacc…3'
CV3124	LTR 5'	nt 533 – nt 552	5'…aatcccggacgagcccccaa…3'
CV3028	*gag*	nt 1263 – nt 1282	5'…agcccaacgccggggatctt…3'
CV3029	*gag*	nt 1357 – nt 1376	5'…cggggccttggacgatggtg…3'
CV3032	*pol*	nt 3616 – nt 3635	5'…ttcctgcggccctttgcctc…3'
CV3033	*pol*	nt 3740 – nt 3759	5'…agcccgccaagagacctgct…3'
CV3038	*env*	nt 5920 – nt 5939	5'…ccagaaccgacgggggcttg…3'
CV3039	*env*	nt 6030 – nt 6049	5'…gctggagatcaccgaggcgg…3'
CV3127	miRNAs	nt 6804 – nt 6823	5'…acgccctgttgcacacccttt…3'
CV3128	miRNAs	nt 6927 – nt 6946	5'…ctcagaacccggggccttgc…3'
CV3044	Tax_{BLV} *E2*	nt 7752 – nt 7771	5'…cttgtggacccccctccggct…3'
CV3045	Tax_{BLV} *E2*	nt 7849 – nt 7869	5'…agggctcgcctaggggtagaa…3'
CV3046	LTR3'	nt 8095 – nt 8119	5'…tggttgctagcgggaaactaagact…3'
CV3047	LTR3'	nt 31 – nt 50 and nt 8014 – nt 8033	5'…ctggtttacggggcggtggc…3'
CV3176	Jonction 3'	nt 423 – nt 442 and nt 8612 – nt 8631	5'…aagggcgtctggcttgcacc…3'
CV3177	Jonction 3'		5'…ggccgtcgagttccgacctg…3'
CV3179	*Rasa3 I2*		5'…tcccgagccctaggtgtgcc…3'
CV3180	*Rasa3 I2*		5'…ctcctctcccgcaggccagt…3'
CV3181	*Rasa3 E2*		5'…tcccacatacccggggccaa…3'
CV3182	*Rasa3 E2*		5'…tggtcctgaaaacctcctcctggt…3'
CV3044	$tRNA^{Asp}$		5'…tcgttagtatagtggtgagtatccccg…3'
CV3045	$tRNA^{Asp}$		5'…ggggaatcgaaccccggtctcc…3'
CV3357	*5S rDNA*		5'…tggccataccaccatgaatgcacc…3'
CV3358	*5S rDNA*		5'…cgatccaagtactaaccaggtccaagc…3'
CV3335	*U6 RNA*		5'…cgcttcacgaatttgcgtgtcat…3'
CV3359	*U6 RNA*		5'…tgctcgcttcggcagcacat…3'
CV3360	*7SK RNA*		5'…ggctaggcgggtgtcccctt…3'
CV3361	*7SK RNA*		5'…tcgtcctcttcgaccgagcg…3'
CV3312	miR-B4-3p	nt 6745 – nt 6757	5'…agcaccacagtct…3'
CV3332	miR-191		5'…cggcggcaacggaaucccaaa…3'
CV3334	miR-B2-5p	nt 6508 – nt 6521	5'…cggcggatgactgagtgtag…3'
CV3313	Stemloop		5'…gtgcagggtccgaggt…3'
CV2564	*Fluc*		5'…cccgcgaacgacatttataa…3'
CV2565	*Fluc*		5'…tttggaaacgaacaccacg…3'
CV2004	*GAPDH*		5'…gcccccggtttctataaattg…3'
CV2005	*GAPDH*		5'…agaagatgcggctgactgtc…3'

Table S3 : List of qPCR primers used in this study
